# The Use of AlphaScreen Technology in HTS: Current Status

**DOI:** 10.2174/1875397300801010002

**Published:** 2008-02-25

**Authors:** Richard M Eglen, Terry Reisine, Philippe Roby, Nathalie Rouleau, Chantal Illy, Roger Bossé, Martina Bielefeld

**Affiliations:** 1 President, Molecular Medicine, PerkinElmer Life and Analytical Sciences, 940 Winter St., Waltham, MA 02451-1457, USA; 2 PerkinElmer, 1744 Rue William, Suite 600, Montreal Qc H3J 1R4, Canada

## Abstract

AlphaScreen (Amplified Luminescent Proximity Homogeneous Assay Screen) is versatile assay technology developed to measuring analytes using a homogenous protocol. This technology is an example of a bead-based proximity assay and was developed from a diagnostic assay technology known as LOCI (Luminescent Oxygen Channeling Assay). Here, singlet oxygen molecules, generated by high energy irradiation of Donor beads, travel over a constrained distance (approx. 200 nm) to Acceptor beads. This results in excitation of a cascading series of chemical reactions, ultimately causing generation of a chemiluminescent signal.

In the past decade, a wide variety of applications has been reported, ranging from detection of analytes involved in cell signaling, including protein:protein, protein:peptide, protein:small molecule or peptide:peptide interactions. Numerous homogeneous HTS-optimized assays have been reported using the approach, including generation of second messengers (such as accumulation of cyclic AMP, cyclic GMP, inositol [1, 4, 5] trisphosphate or phosphorylated ERK) from liganded GPCRs or tyrosine kinase receptors, post-translational modification of proteins (such as proteolytic cleavage, phosphorylation, ubiquination and sumoylation) as well as protein-protein and protein-nucleic acid interactions.

Recently, the basic AlphaScreen technology was extended in that the chemistry of the Acceptor bead was modified such that emitted light is more intense and spectrally defined, thereby markedly reducing interference from biological fluid matrices (such as trace hemolysis in serum and plasma). In this format, referred to as AlphaLISA, it provides an alternative technology to classical ELISA assays and is suitable for high throughput automated fluid dispensing and detection systems.

Collectively, AlphaScreen and AlphaLISA technologies provide a facile assay platform with which one can quantitate complex cellular processes using simple no-wash microtiter plate based assays. They provide the means by which large compound libraries can be screened in a high throughput fashion at a diverse range of therapeutically important targets, often not readily undertaken using other homogeneous assay technologies. This review assesses the current status of the technology in drug discovery, in general, and high throughput screening (HTS), in particular.

## INTRODUCTION

AlphaScreen is a channeling immunoassay in which the immune reaction coordinates two bead components into close proximity on a defined physical surface. At that surface, one component produces a product that serves to activate a second component, thereby producing a detectable signal. In many aspects of drug discovery, principally high throughput screening (HTS), AlphaScreen has been widely deployed in aspects of cell signaling research, drug discovery and biomarker quantification [1-5]. This is wide adoption results from both the simplicity of assay protocols, but also from the high sensitivity of the assay. These two properties, as well as with the fact that wash and separation are not used, allow a range of assays to be configured for automated liquid handling and detection instrumentation frequently used in HTS.

The technology employs oxygen channeling chemistry developed initially as a diagnostic detection assay platform - LOCI [6-7]. The approach exploits the short diffusional distance of singlet oxygen to initiate a chemiluminescent reaction in the proximity of chemical generation. In contrast to other methods based upon light scattering, the method is insensitive to interference by particles or other substances present in biological sample matrices. The assay format comprises two discrete ligand - or receptor - coated polystyrene beads, designated as “Donor” and “Acceptor” beads, which form pairs in the presence of analyte. The pairs must be within about 200 nm (approximately the diameter of a bead particle) in order for a chemiluminescent signal to be generated. Donor beads contain a photosensitizing agent (phthalocyanine) that, when irradiated at 680 nm, excites ambient oxygen to a singlet state. Excitation of each Donor beads generates approximately 60,000 oxygen singlets per second [8], resulting in a highly amplified response upon interaction with Acceptor beads.

**Fig. (1). AlphaScreen/AlphaLISA Assay Principle:**
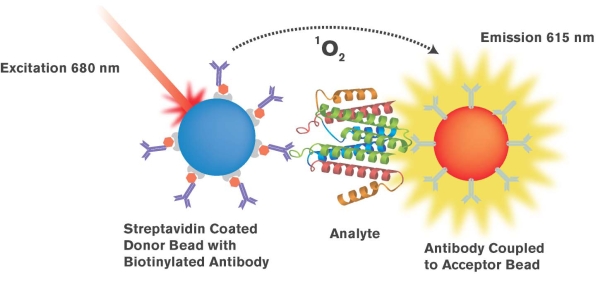
AlphaScreen/AlphaLISA assays are bead based proximity assays, based upon an oxygen channeling technology. When the Donor (blue bead), which contains phthalocyanine, is laser excited (at 680 nm) ambient oxygen is converted to singlet oxygen. This is a highly amplified reaction since approx. 60,000 singlet oxygen molecules can be generated and travel at least 200 nm in aqueous solution before decay. Consequently, if the Donor and Acceptor (gold beads) beads are within that proximity, energy transfer occurs. Singlet oxygen molecules react with chemicals in the Acceptor beads to produce a luminescent response. If the Acceptor bead contains Europium, as in the AlphaLISA assay, an intense luminescence is emitted at a wavelength of 615 nm.

In all AlphaScreen assays, the Acceptor beads contain three chemical dyes; thioxene, anthracene and rubrene (hence the designation, ‘TAR’ beads). Thioxene reacts initially with singlet oxygen to produce light energy, which is subsequently transferred to anthracene and thence to rubrene. The final compound in the cascade, rubrene, emits light at wavelengths of 520-620 nm. Since this process occurs against a very dark background, highly sensitive assays can be developed. Moreover, the bead particles are often present in assays in low concentrations. Consequently, non specific close proximity interactions of Donor and Acceptor particles is rare and again the background is low (Fig. **1**). Since the lifetime of the singlet oxygen reactive species in aqueous solutions is very short (approx. 4 ms), donor and acceptor beads need to be bound (frequently *via* an immunological complex) to one another in order to generate a signal. Those unbound beads experience extremely low singlet oxygen concentrations, and thus contribute minimally to the background signal. Recently, Acceptor beads have been modified to contain Europium, and commercialized as AlphaLISA assays. In AlphaScreen assays using TAR beads the conversion of dioxetane to diketone (due to the reaction of thioxene with singlet oxygen) can be rate limiting. By contrast, AlphaLISA beads, containing Europium, provide an emission that is very highly intense and spectrally defined (615 nm), in comparison to AlphaScreen assays (520 - 620nm).

In both AlphaScreen and AlphaLISA assays, the bead surface are coated with latex-based hydrogels containing reactive aldehydes. This coating both reduces non specific binding but additionally provides a functionalized surface to which a variety of different ligand or receptor binding partners may be affixed. Collectively, these surface properties allow quantitation of a wide range of enzymes, protein-protein interactions, as well as DNA and RNA hybridization. In contrast to the short distances required for resonance energy transfer (RET) approaches, AlphaScreen Donor/Acceptor pairs can be separated by as far as 200 nm for energy to transfer efficiently. The size of the separation distance (which is relatively large by comparison to FRET techniques) allows greater flexibility in the choice of analyte (up to and including large proteins and even phage particles) that can be quantitated.

As mentioned above, AlphaScreen was developed from LOCI technology; a bead based assay technology developed in the mid 1990’s by Ullman and colleagues [6-7] at Syva Diagnostics (later Dade Behring). It was designed to measure clinically important analytes (including cyclosporine, digoxin, estradiol, folate, theophylline or TSH) in plasma or serum and later, tissue nucleic acids [9] (Table **1**). A major advantage of the method was that, even though there were no fluid separation steps in the assay protocol, interference by blood constituents was markedly reduced. Currently, several Svya/Dade Behring LOCI assays continue to be widely used in the *in vitro* diagnostics industry. In 1999, PerkinElmer acquired the exclusive rights to develop LOCI technology for use in research and drug discovery applications. Currently commercialized from this company under the name, AlphaScreen, and now AlphaLISA [1], the technology has been used to measure several signal transduction molecules including cyclic AMP [10-11] and inositol [1, 4, 5] trisphosphate [12], as well as protein kinases [4, 5, 11, 13] and proteases [8, 14]. It has also been used in proteomics [15] and genomics studies in addition to screening at nuclear hormone receptors [3, 16-18], transcription factors [19], and single nucleotide polymorphisms (SNPs) [2, 20, 21].

There is, therefore, a significant literature now available on the use of the technique in many laboratories. This paper assesses the current status of AlphaScreen and AlphaLISA in both basic drug discovery research and HTS.

## ALPHASCREEN ASSAYS FOR HTS

### Second Messenger Accumulation Assays

Almost all HTS assay protocols require sequential addition of reagents. Consequently, sandwich competition immunoassays are frequently used. In the case of AlphaScreen, assay times are minimized by using, as a first reagent addition, antibody-labeled beads as well as a second antibody labeled with biotin. Both reagents have good stability and can be stored together with minimal nonspecific binding prior to use. The second reagent comprises streptavidin-labeled beads, often added in relatively high concentrations. Attaching the second antibody to this bead, as opposed to the inclusion a second antibody with the first reagent, leads to slower rate of binding in the second step. Consequently, in most assay formats, AlphaScreen employs Donor beads coated with streptavidin that bind biotinylated proteins, peptides or small molecules. In the majority of assays, Acceptor beads are coupled to their cognate binding partners optimized to detect the analyte under investigation.

**Fig. (2). Comparative protocols of ELISA vs. AlphaLISA assays:**
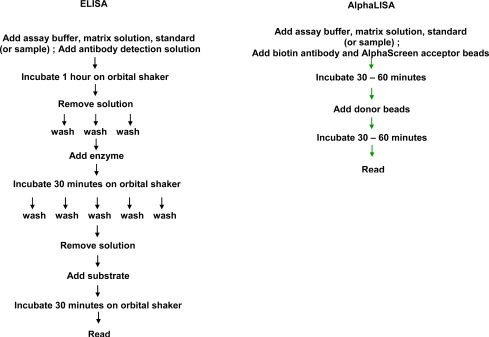
In these schematic protocols, panel on the left illustrates the multiple wash and separation steps required in a typical ELISA experiment. By contrast, the use of the AlphaLISA allows experiments to be conducted using simple mix and read protocols.

AlphaScreen has been used to develop assays to measure cAMP accumulation, particularly for high throughput cell-based GPCR screening [10, 11]. AlphaScreen has also been developed to measure other cyclic nucleotides, such as cGMP, using an analogous format. AlphaScreen cAMP assay employs Donor beads coated with streptavidin in order to bind biotinylated cAMP. The acceptor beads are coated with anti-cAMP antibodies. Bound biotinylated cAMP interacts with anti-cAMP antibodies, bringing Donor and Acceptor beads into close proximity. Singlet oxygen then transfers, resulting in a chemiluminescent signal (Fig. **2**). Addition of the cell lysate containing free cAMP competes with biotinylated cAMP for the antibody, reducing the signal in a concentration-dependent fashion. This assay is usually undertaken using the crude cell lysate following activation of the GPCR.

In modern HTS laboratories, numerous homogeneous assays are now available for the measurement of cAMP accumulation in response to liganded GPCR activation including Time Resolved Fluorescent Energy Transfer (TR-FRET), Fluorescent Polarization (FP), Enzyme Fragment Complementation (EFC) and Electrochemical Luminescence (ECL) techniques. Most provide an order of magnitude in sensitivity in detection such that about 10 femtomolar cAMP per well can be measured. However, it must be noted that AlphaScreen and ECL are chemiluminescent technologies that, whilst robust and cheap, require a specific detection instruments.

Surprisingly few studies have been reported a direct comparison all of these methods. However, Elster *et al*. [23] have recently compared the pharmacology of β_1_-adrenergic receptor ligands, measured by β-arrestin translocation assays *via *bioluminescence resonance energy transfer (BRET), with that obtained using cAMP responses quantified using AlphaScreen. It was found that both techniques detected full agonism and antagonism, yet only the AlphaScreen assay was sensitive enough to detect inverse agonism. However, it is probable that the disparity was due less to the assay methods evaluated, but is more to a greater ‘receptor reserve’ associated with the “downstream” response of cAMP accumulation, in comparison to β-arrestin translocation.

### AlphaScreen Assays for protein kinases

Kinases serve to catalyze the transfer of terminal phosphate groups from ATP to target substrates [24-27]. They comprise two major families - those that catalyze phosphorylation of tyrosine residues and those that phosphorylate either serine or threonine residues. AlphaScreen assays have now been developed to detect the function of both families [4, 5, 13, 28]. In the case of tyrosine kinases, AlphaScreen assays utilize antibodies selective for phosphorylated tyrosine residues. These are directly conjugated to Acceptor beads and biotinylated peptides, acting as kinase substrates, were conjugated to Donor beads. Binding of biotinylated phosphorylated substrates and the phosphoselective antibodies coordinates the Donor and Acceptor beads into close proximity and generates an assay signal (Fig. **2**).

In general, serine/threonine kinases exhibit higher specificity for substrates in comparison to substrates for tyrosine kinases. Consequently, very selective antibodies are required to detect serine/threonine kinase function. To this point, AlphaScreen assays have now been developed using antibodies that specifically detect the phosphorylated substrate. Here, Acceptor beads are usually conjugated to protein A in order to bind mouse or rabbit derived antibodies. Both phosphorylated biotinylated peptides as well as phosphorylated GST-fusion protein substrates can be used to bind to streptavidin or glutathione (GSH) coated Donor beads, respectively. In this format either peptides or proteins kinase substrates, once phosphorylated, bind to the anti-phosphoprotein antibodies, thus generating an assay signal (Fig. **2**).

In a similar fashion to GPCRs, many homogeneous assays are now available for screening at kinases, including TR-FRET, FP, EFC, and ECL. All require selective antibodies to detect the phosphorylated peptide. In the case of serine/threonine kinases, this requirement restricts the type of assay that can be developed. In an attempt to overcome these limitations, TR-FRET, EFC and FP approaches have been adapted to use Lewis metals to coordinate the substrate phospho group, as a surrogate of a phospho specific antibody. Consequently, a similar approach using Lewis metals has also been reported using AlphaScreen [29, 30]. Here, the phosphorylated kinase substrate can be either biotinylated or generated as a GST fusion protein - which is captured by either streptavidin coated or Glutathione coated Donor beads, respectively (Fig. **2**). The acceptor beads are then coated with Lewis metal chelates that act to coordinate substrate phosphate groups. Once phosphorylated GST labeled kinase substrates bind to the Lewis metal chelate, they are then captured by GSH coated Donor beads forming a proximity interaction. Although none of the ‘antibody free methods’ discriminate between multiple phosphorylation events on the same substrate, a major advantage of the AlphaScreen approach, in comparison to TR-FRET, is that both protein or peptide substrates can be used.

**Fig. (3). Surefire assay principle:**
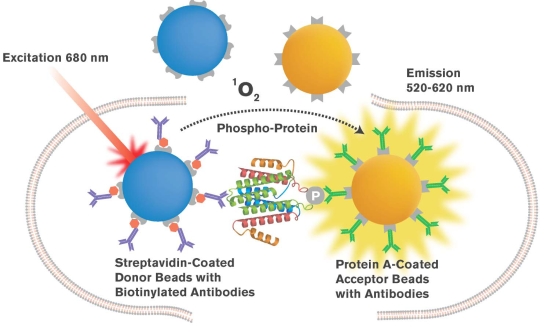
The AlphaScreen platform can be used to detect analytes in a crude cell lysate using protocols developed into the Surefire format. Here, the levels of a phosphorylated protein (such as phosphoERK_1/2_ can be quantified using the approach, by use of specific antibodies to the phosphoprotein, without wash and separation steps as in a typical ELISA assay.

A second assay format often utilized to measure serine/threonine kinase activity employs a “sandwich” immunoassay approach (see Fig. **3**). In this format, an antibody pair is used to detect phosphorylated substrates, in a manner fashion to a classical ELISAs. One antibody is used to detect the form of kinase substrate independent of its phosphorylation state and a distinct, second antibody is used that is specific to the phosphorylated state of the substrate. One of the antibodies is biotinylated and then captured to the bead. Streptavidin coats the Donor beads while the Acceptor beads are labeled with the second antibody in a similar manner to that employed in a typical AlphaScreen IgG assay. This format has the major advantage that it does not require substrate labeling yet provides greater sensitivity than many ELISA based methods.

The assays discussed above measure kinase function as a means to measure the potency of a putative enzyme inhibitor. One problem with the approach is that the kinase under investigation needs to exhibit significant activity (in terms of substrate phosphorylation) to generate a detectable assay signal. However, numerous kinases of clinical importance are expressed with only low activity. A recent development of AlphaScreen technology therefore is to directly measure the potency of the kinase inhibitor *via* its binding constant, using peptide substrates that interact at the kinase active site. Indeed, such peptides may be used as probes that can identify selective inhibitors acting at the substrate binding site. This concept, described in Guenet *et al*. [5] for Jun kinase 3 (JNK 3), involves coupling, *via* a His tag, of the kinase to a nickel ion coated Acceptor bead, together with the conjugation of either the kinase substrate (e.g. cJun) or a peptide inhibitor as a GST fusion protein to the Donor beads (previously coated with an anti-GST antibody). When either c Jun, or a JNK inhibitor peptide, binds to JNK3, a luminescent response is detected *via* a proximity assay.

In most inhibitor programs, most compounds are directed against the ATP binding domain - a site that is highly conserved across kinases. Consequently, attainment of high kinase specificity may be problematic since ATP-site selective compounds possess a degree of cross reactivity to other kinases. By contrast, small molecules that selectively inhibit unique orthogonal *substrate* binding sites are thought to have higher selectivity. Consequently, kinase binding assays are now been emerging as HTS assays and have been reported using both EFC [31] or TR-FRET techniques, in addition to the AlphaScreen approach discussed above.

In the assays described above, most employ cell-free (“biochemical”) formats that mandate the use of highly purified kinases and kinase domains. However, there is now a significant move in screening programs towards assay technologies capable of studying kinase function in the cell - particularly those use homogeneous protocols adaptable to high throughput instrumentation. In this regard, AlphaScreen assays have a unique advantage in that they can be developed to measure kinase phosphorylation of endogenous substrates, including other kinases in the signaling pathway, in the intact cell. Osmond *et al*. [32] described an AlphaScreen assay to measure the phosphorylation of ERK_1/2 _following GPCR activation. This involves antibodies that selectively detect phosphorylated ERK_1/2_ (coordinated by protein A Acceptor beads) and biotinylated antibodies detecting ERK_1/2_ independent of the phosphorylation state (captured by streptavidin donor beads). This approach (commercialized under the term Surefire) can be employed for both GPCR screening as well as for growth factor receptors, such as TNFα receptors (Fig. **3**). Recently, analogous assays have now been developed to measure phosphorylation other kinases, including p38 MAPK, JNK3, MEK, AKT and p70.

### AlphaScreen Assays for proteases

AlphaScreen assays have been reported to measure activity of the protease, ADAM [8]. This enzyme acts to cleave the protein, aggrecan, which is a critical step for cartilage formation and for which disregulation is involved in the etiology of arthritic joint disease. Peppard *et al*. [8] developed a protease assay by coating both Donor and Acceptor beads with antibodies targeting different epitopes of aggrecan, thus allowing the protein to create a bridge between the beads. A luminescent response was then detected only when full length protein substrates were present. In the presence of the protease, ADAM, the Aggrecan Bridge is hydrolyzed and the luminescent signal reduced. The assay was used to screen a library of small molecules in an HTS format. Although many HTS technologies are now available to measure protease activity, several specific advantages are uniquely conferred by an AlphaScreen approach [8], including the ability to use much larger substrates than can be utilized in traditional FRET-based assays. Moreover, Peppard *et al*. [8] used essentially four antibodies and the large molecular weight aggrecan to bridge the Donor/Acceptor bead complex. Using full length protein substrates has the advantage in that several proteases possess multiple cleavage sites. Employing peptide substrates that encompass only single cleavage sites will miss critical protease function detectable only with the use of large protein substrates.

### Ubiquitin Ligase AlphaScreen Assays

Many cellular proteins undergo ubiquitination; a post-translational modification that “targets” proteins for degradation in the proteosome. This modification is mediated by the E3 ligase family and which is therefore essential for normal protein turnover. Consequently, disregulation of E3 ligase activity causes pathologies such as Alzheimer’s and Parkinson’s disease, several cancers, and inflammatory diseases. Despite the importance of ubiquitin ligases as therapeutic targets, most current HTS approaches at these targets are low in throughput and primarily employ immunoblotting techniques. Kus *et al*. [33] have developed an E3 ligase assay using AlphaScreen technology to identify novel substrates that may undergo ubiquitination. This group screened the yeast E3 ligase, Rsp5, against a large number of GST tagged protein substrates to identify optimal Rsp5 substrates. The assay utilizes the transfer of biotinylated ubiquitin to target substrates by Rsp5, resulting in the production of biotinylated and ubiquitinated GST tagged proteins. The biotin tag present on the ubiquitinated GST fusion protein allowed its simultaneous capture by streptavidin Donor and anti-GST /Acceptor beads resulting in signal generation. Kus *et al*. [33] identified several substrates for Rsp5 including many unknowns. Subsequently, the ubiquitination of these substrates was confirmed in orthogonal assays using gel electrophoresis and cellular toxicity assays.

### Protein-Protein Interaction Assays Using AlphaScreen

Many cellular reactions involve complex protein-protein interactions, ranging from ligand/ GPCR binding, G protein coupling, interaction of kinases with cognate substrates as well as the interaction of transcription factors with nuclear co-activators and co-repressors. Partly due to this cellular complexity, few HTS technologies have been developed that measure protein-protein interactions that can be used to identify either small molecule inhibitors or novel interacting partners. In this respect AlphaScreen technology has emerged as a useful assay platform to measure protein: protein interactions, as will be discussed below.

#### Growth Factor Receptor Binding

Tumor necrosis factor (TNFα) receptors are implicated in the etiology of many inflammatory and immunological disorders. Wilson *et al*. [17] developed an AlphaScreen assay to measure ligand binding to a member of the TNF receptor super-family, OX40 (CD 134). A fusion protein was generated in which OX40 was coupled to a domain of human IgG, allowing OX40 to bind to Acceptor beads coated with protein A. An OX40 ligand (OX40L-CD8) tagged with biotin which was coordinated by streptavidin coated Donor beads. The binding of OX40L to OX40 bridged of Acceptor/Donor beads and therefore generated a signal. Therefore, OX40 ligand binding could directly measured in a HTS format and subsequently, several peptides and small molecule were identified that inhibited OX40L binding.

#### Transcription Factors

AlphaScreen technology has also been adapted to measure the interaction of various transcription factors with nuclear binding sites. Rouleau *et al*. [3] have reported an AlphaScreen assay to measure interactions between estradiol-bound estrogen receptors (ER) and the steroid receptor coactivator 1 (SRC-1). ER was coordinated to Acceptor beads coated with anti-ER antibodies and a peptide fragment (derived from SRC-1 sequences) was biotinylated and coupled to streptavidin coated donor beads. ER agonists induced the interaction of ER with SRC-1 and resulted in an assay signal. The assay exhibited pharmacological characteristics similar to that observed using other technologies. An equivalent assay for the retinoic acid receptor was also developed and again exhibited appropriate pharmacological specificity. Collectively, these studies indicated that AlphaScreen technology could be used to identify novel ligands and inhibitors acting at nuclear receptor binding sites in high throughput assay protocols.

#### Viral Protein Binding

An important molecular interaction with regard to infectious disease and oncology is the mechanism by which viral proteins induce cell transformation and proliferation. For example, Human papilloma virus (HPV) is a major cause of cervical cancer. HPV induces cell transformation in part by inhibiting the activity of p53, and by blocking transformed cells from undergoing apoptosis. These effects result from an interaction of HPV protein E6 with the ubiquitin ligase, E6AP. Consequently, small molecules that inhibit the binding of E6 to E6AP may attenuate the ability of HPV to cause cervical cancers. Sehr *et al*. [34] reported an AlphaScreen assay to measure E6 binding to a peptide fragment of E6AP and used the assay to detect inhibitors of the interaction. Here an E6-GST fusion protein was bound to Acceptor beads coated with either anti-GST antibodies or GSH. A biotinylated peptide fragment of E6AP, known to be a site of interaction of E6 was synthesized and bound to Streptavidin coated Donor beads. Using this approach, Sehr *et al*. [34] screened approximately 3000 small molecules and identified several leads that were validated in subsequent screens.

## ALPHALISA

Currently, most biomarker analysis requires high capacity detection of analytes as markers of biomolecular reactions. The scale of high throughput analysis requires detection instrumentation and assay platforms easily adapted to automated systems. A standard approach used for many years in biomarker analysis is ELISA technology. Although conventional ELISAs readily detect analytes in the low picomolar (pM) range, they have several limitations that restrict their use in high throughput protocols (Table **2**). Specifically, ELISAs require multiple washing steps to remove non-specifically adsorbed reactants (Fig. **2**). These are both time consuming and require complex instrument programming that serve to increase assay costs and decrease precision. Although ELISAs handle relatively low sample volumes adequately, larger volumes are usually needed due to limitations in assay sensitivity. This is restrictive in screens where the target analyte is present only in low concentrations. Furthermore, many ELISAs often exhibit approximately two orders of magnitude in dynamic range. Consequently, the sample has to be diluted several times to bring analytes levels within the range of ELISA detection. A comparison of AlphaScreen /AlphaLISA platforms with other assay formats are shown in Table **3**.

The AlphaLISA platform is an evolution of the AlphaScreen technology designed to meet requirements for high throughput assays for biomarker detection. AlphaLISA resembles AlphaScreen, except that the beads contain Europium as the Acceptor fluor. Europium emission is more intense and better defined spectrally (615 nm) than observed in AlphaScreen assays (520-620nm). Consequently, the emission wavelength is less prone to matrix interferences from compounds such as hemoglobin or transferrin. AlphaLISA assays also require small sample volumes (5 μl) yet they have over a 100-fold greater analytical range than ELISA. Since AlphaLISA can employ the same antibody pairs assays using this approach exhibits equivalent selectivity to ELISAs but are more sensitive due to its highly amplified nature.

Poulsen and Jensen [15] have recently reported direct comparisons of AlphaLISA and ELISA assays to measure human insulin in plasma samples. The assay sensitivity was shown to be in the sub-picomolar range with a ten thousand fold dynamic range of detection. The assay also had excellent intra- and inter-assay precision and was unaffected by plasma and serum matrices. The samples were then simultaneously analyzed using the AlphaLISA and a standard ELISA method using the same antibody pairs and a good correlation between the two was seen. Furthermore, AlphaLISA detected 15-fold lower levels of analyte than ELISA, while using one fifth the sample volume. It also had over 100-fold greater assay range with similar precision levels. The authors thus concluded that AlphaLISA platform provided a viable alternative to ELISA and being homogenous was more amenable to automated fluid dispensing systems.

Several other technologies, besides AlphaLISA, have also been developed as ELISA alternatives. Two technologies to note are the homogenous fluorometric microvolume assay technology (FMAT) [35] and ECL [36]. FMAT can be formatted such that biotinylated antigen interacts with streptavidin coated beads. An AlexaFluor-647 tagged antibody binds to antigen and a luminescent signal is emitted from aggregated beads. Specific signal is then discerned from background using laser-scanning microscopy. In an ECL assay, biotinylated antigen is bound to Streptavidin coated electroplates and binding of ruthenium-labeled anti-mouse antibodies in close proximity to an electroplate results in a chemiluminescent signal. Like AlphaLISA, FMAT and ECL technologies are highly sensitive, do not require extensive washing and can be formatted for HTS. Beasley *et al*. [37] have recently preliminary data that compared FMAT, ECL and AlphaLISA methods to detect common biomarkers. Here, AlphaLISA exhibited higher sensitivity and required lower sample volumes than the other assays. They also indicated that ECL and FMAT techniques involved the use of specialized detection instrumentation (laser scanning microscopy for FMAT) and equipment (electrochemical plates for ECL) which limited their flexibility yet increased assay cost. ECL was also limited by the high costs of the consumable electrochemical plates required.

## SUMMARY

As with any assay technology there are advantages and limitations and this is also extends to AlphaScreen/AlphaLISA assays. The main disadvantage of AlphaScreen technology is that it is sensitive to intense light or long exposure to ambient light. Although this issue was reported in the original publications describing LOCI technology, the problem is now easily overcome by simple assay adjustments. In addition, singlet oxygen can be sequestered by compounds in screening libraries that can scavenge radical oxygen. Donor bead photo bleaching can be a limitation in that the system is effectively limited to a single read. Although AlphaScreen has greater flexibility than technologies such as ECL and FMAT, all three require a high-energy laser excitation source. Consequently, Alphascreen is not adaptable to all readers and in this respect is more limited than other luminescent technologies.

The main advantage of the technology is that it is applicable to a very broad range of analytes, some of which have been addressed in this review. All the assays are homogeneous, rapid, and robust yet appear to be more sensitive than previously reported immunoassay methods. Furthermore, AlphaScreen assays do not require insertion of large fluorescent epitope tags that may sterically hinder bio molecular interactions. AlphaScreen and notably AlphaLISA can be employed to measure enzyme activity in crude biological fluid extracts such as cell lysates, serum and plasma and a variety of cellular/body fluid matrices that do not easily affect the assay readout.

## CONCLUSION

Both AlphaScreen and AlphaLISA assays can be used to measure a diverse range of molecular interactions of interest throughout drug discovery. The homogenous nature of the technique allows it to be an important tool in high throughput screening of new small molecules and, most recently, of novel protein therapeutics. For example, it has been used for hybridoma-screening for thousands of clones that express antibodies for therapeutic development. Such screening presently involves use of ELISAs which, as noted above, are less adaptable for high capacity screening and potentially more costly.

The ability of AlphaScreen to measure post-translational protein modification offers novel uses in screening. Although measurement of second messenger accumulation has been a major application of AlphaScreen technologies, these processes can now also be measured by alternative technologies. However, other cell signaling events such as phosphorylation, proteolysis, ubiquitination, sumoylation and glycosylation, remain difficult to measure. AlphaScreen technology has now been used to successfully screen against these processes. Consequently, novel small molecules or biotherapeutics may be identified to treat disorders such as Alzheimer’s, Parkinson’s and Huntington’s disease, as well as oncology and various immunological disorders.

## Figures and Tables

**Table 1:** Timeline for Development of AlphaScreen Technology

1994 - LOCI developed by Syva/Dade Behring to measure clinical analytes in human fluids [6,7]
1999 - LOCI commercialized for nucleic acid detection in miniaturized formats [9]
1999 - LOCI technology licensed by PerkinElmer for drug discovery applications [1]
2001 - AlphaScreen HTS cAMP assay [10,11]
2001 - AlphaScreen assay for single nucleotide polymorphism (SNP) detection [2, 20, 21]
2002 - AlphaScreen Nuclear Receptor Assay 3, [16, 18]
2003 - AlphaScreen Ins P_3_ assay [12]
2003 - AlphaScreen Protease assay [8, 14]
2003 - AlphaScreen protein kinase assays [11, 13]
2005 - AlphaScreen Transcription factor assays [19]
2005 - AlphaScreen Ubiquitin ligase assay [33]
2006 - Cell based kinase assays using Surefire (AlphaScreen) technology [5]
2006 - Homogeneous ELISA (AlphaLISA) assays [15, 37]

**Table 2:** Comparison of AlphaLISA with ELISA Techniques

	ELISA	AlphaLISA
**Selectivity**	High	High
**Sensitivity**	Sensitive (pM range)	Sensitive (sub-pM)
**Assay Format**	Heterogeneous	Homogenous
**Antibody req.**	Requires matched antibody pairs	Used with antibody pairs
**Assay vol.**	large (25-50 µl)	small (≥ 5 µl)
**Dynamic range**	limited (~2 orders of mag.)	large (~4 orders of mag.)
**Instrumentation**	any luminescent reader	requires high energy laser

**Table 3:** Comparison of AlphaScreen/AlphaLISA Platforms with LANCE and DELFIA

	AlphaScreen/AlphaLISA™	LANCE	DELFIA
**Detection**	Luminescence Proximity	TR-FRET	TRF
**Homogenous**	Yes	Yes	No
**Throughput**	High	Ultra-High	Medium
**Automation**	****	****	*
**Sensitivity**	****	***	****
**Dynamic range**	2.5 - 5 logs	2 - 3 logs	2.5 - 5 logs
**Microplate formats**	96, 384, 1536	96, 384, 1536	96, 384
**Multiplexing**	No	No	up to 4-plex
**Substrate sizes**	Small molecules to whole cells	Small molecules to peptides	Small molecules to proteins,
**Use of low affinity antibodies**	Yes	Limited; impact on data quality	No
**Use of polyclonal antibodies**	Yes	Limited; only special affinity purified Abs	Yes
**Reader**	EnVision	EnVision, Victor, ViewLux & others	EnVision, Victor, ViewLux & others

## References

[1] Bosse R, Illy C, Elands J, Chelsky D (2000). Miniaturizing screening: How low can we go today?. Drug Discov. Today HTS supplement.

[2] Beaudet L, Bedard J, Breton B, Mercuri RJ, Budarf ML (2001). Homogeneous assays for single-nucleotide polymorphism typing using AlphaScreen. Genome Res.

[3] Rouleau N, Turcotte S, Mondou MH, Roby P, Bosse R (2003). Development of a versatile platform for nuclear receptor screening using AlphaScreen. J Biomol Screen.

[4] Warner G, Illy C, Pedro L, Roby P, Bosse R (2004). AlphaScreen kinase HTS platforms. Curr Med Chem.

[5] Guenat S, Rouleau N, Bielmann C, Bedard J, Maurer F, Allaman-Pillet N, Nicod P, Bielefeld-Sevigny M, Beckmann JS, Bonny C, Bosse R, Roduit R (2006). Homogeneous and nonradioactive high-throughput screening platform for the characterization of kinase inhibitors in cell lysates. J Biomol Screen.

[6] Ullman EF, Kirakossian H, Singh S, Wu ZP, Irvin BR, Pease JS, Switchenko AC, Irvine JD, Dafforn A, Skold CN, Wagner DB (1994). Luminescent oxygen channeling immunoassay: measurement of particle binding kinetics by chemiluminescence. Proc Natl Acad Sci USA.

[7] Ullman EF, Kirakossian H, Switchenko AC, Ishkanian J, Ericson M, Wartchow C, Pirio M, Pease J, Irvin B, Singh S, Singh R, Patel R, Daffon A, Davalian D, Skold C, Kuran N, Wagner D (1996). Luminescent oxygen channeling assay (LOCI): sensitive, broadly applicable homogeneous immunoassay method. Clin Chem.

[8] Peppard J, Glickman F, He Y, Hu SI, Doughty J, Goldberg R (2003). Development of a high-throughput screening assay for inhibitors of aggrecan cleavage using luminescent oxygen channeling (AlphaScreen). J Biomol Screen.

[9] Patel R, Pollner R, de Keczer S, Pease J, Pirio M, DeChene N, Dafforn A, Rose S (2000). Quantification of DNA using the luminescent oxygen channeling assay. Clin Chem.

[10] Gabriel D, Vernier M, Pfeifer MJ, Dasen B, Tenaillon L, Bouhelal R (2003). High throughput screening technologies for direct cyclic AMP measurement. Assay Drug Dev Technol.

[11] Li Y, Cummings RT, Cunningham BR, Chen Y, Zhou G (2003). Homogeneous assays for adenosine 5'-monophosphate-activated protein kinase. Anal Biochem.

[12] Gray A, Olsson H, Batty IH, Priganica L, Peter Downes C (2003). Nonradioactive methods for the assay of phosphoinositide 3-kinases and phosphoinositide phosphatases and selective detection of signaling lipids in cell and tissue extracts. Anal Biochem.

[13] Von Leoprechting A, Kumpf R, Menzel S, Reulle D, Griebel R, Valler MJ, Buttner FH (2004). Miniaturization and validation of a high-throughput serine kinase assay using the AlphaScreen platform. J Biomol Screen.

[14] Hamilton AC, Inglese J, Ferrer M (2003). A PDZ domain-based assay for measuring HIV protease activity: Assay design considerations. Protein Sci.

[15] Poulsen F, Jensen KB (2007). A Luminescent Oxygen Channeling Immunoassay for the Determination of Insulin in Human Plasma. J Biomol Screen.

[16] Glickman JF, Wu X, Mercuri R, Illy C, Bowen BR, He Y, Sills M (2002). A comparison of ALPHAScreen, TR-FRET, and TRF as assay methods for FXR nuclear receptors. J Biomol Screen.

[17] Wilson J, Rossi CP, Carboni S, Fremaux C, Perrin D, Soto C, Kosco-Vilbois M, Scheer A (2003). A homogeneous 384-well high-throughput binding assay for a TNF receptor using AlphaScreen technology. J Biomol Screen.

[18] Xu HE, Stanley TB, Montana VG, Lambert MH, Shearer BG, Cobb JE, McKee DD, Galardi CM, Plunket KD, Nolte RT, Parks DJ, Moore JT, Kliewer SA, Willson TM, Stimmel JB (2002). Structural basis for antagonist-mediated recruitment of nuclear co-repressors by PPARα. Nature.

[19] Wu X, Glickman JF, Bowen BR, Sills MA (2003). Comparison of assay technologies for a nuclear receptor assay screen reveals differences in the sets of identified functional antagonists. J Biomol Screen.

[20] SyvÃnen A-C (2001). Accessing genetic variation: Genotyping single nucleotide polymorphisms. Nat Rev Genet.

[21] Tsuchihashi Z, Dracopoli NC (2002). Progress in high throughput SNP genotyping methods. Pharmacogenom J.

[22] Williams C (2004). cAMP detection methods in HTS: selecting the best from the rest. Nat Rev Drug Discov.

[23] Elster L, Elling C, Heding A (2007). Bioluminescence Resonance Energy Transfer as a Screening Assay: Focus on Partial and Agonism. Journal of Biomolecular Screening.

[24] Nishzuka Y (1992). Intracellular signaling by hydrolysis of phospholipid and activation of protein kinase C. Science.

[25] Edelman G, Blumenthal D, Krebs E (1987). Protein serine/threonine kinases. Annu Rev Biochem.

[26] Fantl W, Johnson D, Williams LT (1993). Signaling by receptor tyrosine kinases. Ann Rev Biochem.

[27] Yarden Y, Ulrich A (1988). Growth factor receptor tyrosine kinases. Ann Rev Biochem.

[28] Burns S, Travers J, Collins I, Rowlands MG, Newbatt Y, Thompson N, Garrett MD, Workman P,  Aherne W (2006). High-Throughput Screen Identification of Small-Molecule Inhibitors of Protein Kinase B (PKB/AKT) in an AlphaScreen^TM^. J Biomol Screen.

[29] Corthals GL, Aebersold R, Goodlett DR (2005). Identification of phosphorylation sites using microimmobilized metal affinity chromatography. Methods Enzymol.

[30] Moser K, White FM (2006). Phosphoproteomic analysis of rat liver by high capacity IMAC and LC-MS/MS. J Proteome Res.

[31] Vainshtein I, Silveria S, Kaul P, Rouhani R, Eglen RM, Wang J (2002). A high-throughput, nonisotopic, competitive binding assay for kinases using nonselective inhibitor probes (ED-NSIP^TM^). J Biomol Screen.

[32] Osmond RI, Sheehan A, Borowicz R, Barnett E, Harvey G, Turner C, Brown A, Crouch MF Dyer AR (2005). GPCR screening *via* ERK 1/2 : A novel platform for screening G protein-coupled receptors. J Biomol Screen.

[33] Kus B, Gajadhar A, Stanger K, Cho R, Sun W, Rouleau N, Lee T, Chan D, Wolting C, Edwards A, Bosse R, Rotin D (2005). A high throughput screen to identify substrates for the ubiquitin ligase Rsp5. J Biol Chem.

[34] Sehr P, Pawlita M, Lewis J (2007). Evaluation of Different Glutathione S-Transferase– Protein Captures for Screening E6/E6AP Interaction Inhibitors Using AlphaScreen®. J Biomol Screen.

[35] Toney JH, Ogawa A, Blair M, Park W-W (2003). A “mix and read” assay for insulin using fluorometric microvolume assay technology. Assay Drug Dev Technol.

[36] Golla R, Seethala R (2004). A sensitive, robust high-throughput electrochemiluminescence assay for rat insulin. J Biomol Screen.

[37] Beasley JR Swanson R Yang J Dunn D Conversion of ELISA to No Wash Technologies Conference Poster PST1E007, SBS Meeting Montreal 2007 4

